# Introducing a Novel, Broad Host Range Temperate Phage Family Infecting *Rhizobium leguminosarum* and Beyond

**DOI:** 10.3389/fmicb.2021.765271

**Published:** 2021-11-09

**Authors:** Sam Ford, Sara Moeskjær, Peter Young, Rosa I. Santamaría, Ellie Harrison

**Affiliations:** ^1^Department of Animal and Plant Sciences, University of Sheffield, Sheffield, United Kingdom; ^2^Department of Biology, University of York, York, United Kingdom; ^3^Centro de Ciencias Genómicas, Universidad Nacional Autónoma de México, México City, Mexico

**Keywords:** prophage, temperate phage, rhizobia, phage ecology, phage evolution

## Abstract

Temperate phages play important roles in bacterial communities but have been largely overlooked, particularly in non-pathogenic bacteria. In rhizobia the presence of temperate phages has the potential to have significant ecological impacts but few examples have been described. Here we characterize a novel group of 5 *Rhizobium leguminosarum* prophages, capable of sustaining infections across a broad host range within their host genus. Genome comparisons identified further putative prophages infecting multiple *Rhizobium* species isolated globally, revealing a wider family of 10 temperate phages including one previously described lytic phage, RHEph01, which appears to have lost the ability to form lysogens. Phylogenetic discordance between prophage and host phylogenies suggests a history of active mobilization between *Rhizobium* lineages. Genome comparisons revealed conservation of gene content and order, with the notable exception of an approximately 5 kb region of hypervariability, containing almost exclusively hypothetical genes. Additionally, several horizontally acquired genes are present across the group, including a putative antirepressor present only in the RHEph01 genome, which may explain its apparent inability to form lysogens. In summary, both phenotypic and genomic comparisons between members of this group of phages reveals a clade of viruses with a long history of mobilization within and between *Rhizobium* species.

## Introduction

Bacteriophages (phages) are ubiquitous in bacterial populations and contribute significantly to the ecology and evolution of microbial communities. Temperate phages—phages capable of integrating into the bacterial genome—in particular, occupy a multitude of roles in bacterial communities (Harrison and Brockhurst, 2017); like purely lytic phages they can infect and kill susceptible bacteria, contributing to population turnover and driving selection for resistance (Paterson et al., 2010). As integrated phages—“prophages”—they can provide competitive advantages to their hosts—which are known as “lysogens” (Brown et al., 2009); Lytic phage particles produced from the lysogen population at low rates infect and kill non-lysogens—likely to be unrelated competitors—and thus act as anti-competitor “weapons” during competition. Self-exclusion mechanisms encoded by phages mean that lysogens are themselves immune to infection and thus benefit from the resulting competitive release (Brown et al., 2009; Burns et al., 2015). Phages also play a major role as agents of horizontal gene transfer (Harrison and Brockhurst, 2017) either through transduction—packaging and transporting host DNA within viral particles—or by carriage of bacterial genes on the phage genome. Genes encoded on temperate phages can have significant ecological effects, such as the shiga toxin genes encoded on STX phages that make Shigella bacteria highly virulent (Allison, 2007) or photosynthesis genes encoded on Prochlorococcus phages (Lindell et al., 2004). Thus temperate phages can be significant players in microbial ecosystems. For many bacterial groups, however, particularly in terrestrial environments, very little is known about the lives of these genetic symbionts.

Rhizobia—bacteria that form nitrogen-fixing mutualistic symbioses with legumes—are key members of the soil community. Rhizobia are characterized by their ability to form beneficial intracellular infections in legume hosts which form specialized root structures—“nodules”—to house their bacterial symbionts. Here atmospheric nitrogen is fixed and exchanged with the plant for sugars. Rhizobia-mediated nitrogen fixation is a major source of accessible nitrogen for the biosphere (Vitousek et al., 2002). The rhizobia symbiosis is highly dependent on mobile genetic elements. Rhizobia typically contain numerous plasmids and integrated elements, including those encoding genes required for legume symbiosis (Cavassim et al., 2020). Lytic rhizobia phages have been well described and evidence suggests that they may play a role in structuring rhizobia communities, and correspondingly, in interactions with their legume hosts. Rhizobia phages generally show specificity within their host clades (Shahaby et al., 2014) but can have broad infectivity within genera (Msimbira et al., 2016). Phage resistance and susceptibility can lead to differential nodulation success demonstrating a direct role in the important legume symbiosis (Evans et al., 1979; Hashem and Angle, 1988; Ahmad and Morgan, 1994). Lytic rhizobia phages have also been shown to be capable of efficient horizontal gene transfer by transduction (Buchanan-Wollaston, 1979; Finan et al., 1984). However, the presence and role of temperate phages in rhizobia is less well understood.

Currently, only a small number of prophages have been described across rhizobia clades; in *Rhizobium* (Halmillawewa et al., 2015), *Ensifer* (Dziewit et al., 2014; Decewicz et al., 2017), and *Bradyrhizobium* (Dhar et al., 2012). Here we describe a clade of novel temperate phages infecting *Rhizobium leguminosarum* strains nodulating clover from across Europe. Sequence comparisons show that this group is closely related to a lytic phage isolated in Mexico on *R. etli*, RHEph01 (Santamaría et al., 2014), as well as a range of putative prophages detectable within published *Rhizobium* genomes. Characterization of these phages showed that all prophages were “active”—able to form infectious phage particles and new lysogenic infections, with potential implications for host bacteria. Confirming the results of Santamaría et al. (2014), we find that related phage RHEph01 was unable to form lysogens, and genome analysis suggests that this may be an acquired trait rather than a loss of function through gene loss or disruption.

## Materials and Methods

### General Bacterial and Phage Culturing

All bacterial culturing was performed in TY media (3 g/L yeast extract, 6 g/L tryptone, 0.5 g/L calcium chloride) in 6 ml culture volumes under shaken (180 rpm) conditions at 28°C. Colony forming units (CFUs) were enumerated on solid TY agar (13 g/L) and plaque forming units (PFUs) on soft TY agar overlays after cells were removed through filtration (using 0.45 μM syringe filters). Soft agar overlays consist of 5 ml of 6 g/L agar containing 100 μl of a growing culture of phage free *R. leguminosarum* bv. *trifolii* (*Rlt)* TRX19. Where stated phage lysis was induced with mitomycin C. A 1000X concentration of mitomycin C was added at a final concentration of 0.05 μg/ml to lysogenic cultures grown for 6 h. Cultures were then allowed to grow for a further 40 h before phages where harvested. Phage clones were isolated by “picking” a plaque with a sterile toothpick and inoculating into a culture of phage free *R. leguminosarum* bv. *trifolii* (*Rlt)* TRX19.

### Phage Isolation and Sequencing of vTRX32-1

The phage vB_RleA_TRX32-1 (hereafter shortened to vTRX32-1, accession number MW023914.1) was isolated through induction from lysogenic strain, *Rlt* TRX32. The strain was isolated in York, United Kingdom by Kumar et al. (2015) from a clover (*Trifolium repens)* nodule. vTRX32-1 was then cultured on phage-free strain *Rlt* TRX19. Phage clones were amplified from single plaques and DNA extracted from lytic phage particles following published methods (Jakočiūnė and Moodley, 2018). Briefly, 1 ml of a filtered phage lysate was prepared first by digestion of any contaminating bacterial DNA and RNA with DNaseI and RNaseA, followed by lysis of the phage capsid by proteaseK digestion. DNA was then extracted using a DNeasy Blood and Tissue kit (Qiagen). Further purification was achieved using the DNA Clean and Concentrator kit (Zymo) following kit protocol. Phage DNA was sequenced on Illumina MiSeq platform. An initial alignment was performed using SPAdes creating one primary contig of 42.8 Kb (initial coverage of 29.27x). All reads were then realigned to this primary contig using BWA and SAMtools resulting in 88% of reads being mapped and a final mean coverage of 49.1x. The assembly pipeline is provided in Supplementary Materials. Open reading frames (ORFs) were predicted and annotated provisionally using the RAST pipeline (Aziz et al., 2008). The PHASTER webserver (Arndt et al., 2016) and blastp were then used to aid the annotation of the remaining hypothetical ORFs. tRNAs were identified using tRNAscan-SE 2.0 (Chan and Lowe, 2019). Host and phage promoter sequences were predicted using PhagePromoter (Sampaio et al., 2019).

A nucleotide Blast revealed closely related phage sequences in four published bacterial genomes belonging to an extensive (196 strain) collection of *Rlt* strains (Cavassim et al., 2020); SM48, SM95, SM113 and SM135A. These strains were kindly provided by Stig U. Andersen and phages induced as above. Phage clones were isolated from single plaques and verified using phage specific primers. Phages were named based on isolating strain as before, vB_RleA_vSM48-1, or “vSM48-1” from SM48 etc.

The blast search also revealed that these phages are related to the lytic phage, RHEph01, which was isolated on *R. etli* in Mexico and has been previously described (Santamaría et al., 2014; Supplementary Table 1). Phage RHEph01 was kindly provided by Victor Gonzalez for phenotypic comparison with the *Rlt* phages.

### Electron Microscopy

Phage lysates of vTRX32-1 and RHEph01 were prepared through lysis of the mutually susceptible host TRX19. 20 μl of phage stocks were introduced to 1 ml culture in mid-exponential phase. Phages were recovered by filtration with a 0.45 μm syringe filter. A 5 μl spot of phage lysate was added to a copper/palladium grid, left for 1 min and excess removed. Phages were then washed twice in water and stained twice with 1% uranyl formate for 20 s. Grids were examined with a CM100 Transmission electron microscope.

### Phenotypic Comparisons Between Strains

Phage phenotypes were compared for host range, virulence and lytic growth dynamics. Host range was determined for the five *Rlt* phages on a range of hosts drawn from the same culture collections as the original lysogens as well as a number of non-*R. leguminosarum* type strains. The lytic phage RHEph01 was included for comparison. Phages were standardized to 10^6^ PFU per ml and 10-fold serial dilutions spotted onto bacteria grown in soft TY agar overlays. Phages that showed a zone of partial (turbid) clearing at high phage titer after 1 day were graded as “weak.” Phages that produced a clear zone of clearing at high titer and individual plaques at lower densities at day 1 were graded as “strong.” Zones of clearing were inspected each day for a further 3 days. Where regrowth was observed within the high titer spots, bacteria from this turbid zone was streaked out to isolate individual colonies to check for lysogenization. Putative lysogens were confirmed through colony PCR targeting the phage DNA maturase gene (forward; GTCGAGTGCTTGACCTCCTC, reverse: ACCTCTTCTTGGTCGCTTCA) and experimentally by spotting supernatant onto susceptible host TRX19.

Replication dynamics of 3 phages, vTRX32-1, vSM113-2 and lytic phage RHEph01, were determined by one-step growth curves of lytic viral infections. vSM113-2 was selected as the most divergent phage from vTRX32-1. Exponentially growing cultures (*n* = 4) were mixed with phages (100:1) and left to adsorb for 15 min. Unadsorbed phages were then removed and plated to determine the number of phage infections (total phage—unadsorbed). Infected cells were then diluted 10 fold and incubated at 28°C. Samples were taken every 30-min for 8 h and phage density enumerated by plating onto soft agar overlays. Phage growth curves were modeled using R package “GrowthRates” to estimate latent period (time between adsorption and the beginning of phage lysis) and burst size (maximum phages produced per infection).

Virulence of these 3 phages was estimated on susceptible *Rlt* host TRX19 as well as *R. etli* strain Bra5, the original isolating strain of RHEph01. Mid-exponential phase cultures of host strains were diluted 10 fold and combined in a 96 well plate with phage inoculants at a multiplicity of infection 0.1 or phage free TY for a phage- control. Three replicates of each phage x host combination were established and grown for 72 h at 28°C with shaking in an incubated plate reader measuring OD600 at 20 min intervals. Phage virulence was measured as reduction in bacterial growth (RBG) at 2-time points: at 10 h post-infection to estimate the initial impact of infection, and at 40 h, to estimate the overall impact of phage infection on growth (thus capturing both lysis and recovery following in emergence of resistance/lysogenization). RBG was estimated as 1—(ODphage + T_*x*_—ODphage + T_0_)/(ODphage- T_*x*_—ODphage- T_0_), where time = T. Phenotypic data can be found in Supplementary Table 2.

### Genome Annotation and Comparison

In addition to the isolated and previously described phages, four further, more distantly related, prophage sequences were identified in other *Rhizobium* strains through NCBI blastn using the vTRX32-1 genome and default settings: *R. anhuiense* CCBAU 23252, *R. loessense* CGMCC 1.3401, *Rhizobium* sp. NXC14 and *Rhizobiales* sp. AFS092257. These were included for comparison of the wider clade.

Provisional prophage sequences for the four *Rlt* prophages vSM48-1, vSM95-2, vSM113-1 and vSM135A-2, and for the four putative prophages from *Rhizobium* spp. sequences were obtained using PHASTER (Arndt et al., 2016)^1^. Prophage termini (attL and attR sites) and the corresponding integration site in the bacterial genome (attB) were identified based on their alignment to the circular vTRX32-1 and RHEph01 phage genomes and annotation by PHASTER (Arndt et al., 2016) and comparison to phage free *Rhizobium leguminosarum* sequences. A conserved terminally repeated 12 bp motif (“CAGATTTAGGTT”) was identified at the terminal ends of all prophages, which could also be found in phage free strains within a tRNA-leu ORF.

Pairwise blastp of RAST-predicted coding sequences and blastn of whole genomes were used to generate percent identity values between the genes/genomic regions of vTRX32-1 and those of its relatives. Average nucleotide identity was calculated using the VIRIDIC phage genome comparison tool (Moraru et al., 2020)^2^.

### Phylogenetic Analyses of Prophage/Phage

As phage genomes can be highly variable the phage phylogeny was generated using a 5 gene concatenated nucleotide sequence of the RNA polymerase, DNA polymerase, DNA helicase, Collar protein and Maturase B genes. Host phylogeny was produced based on core gene *rpoB*. A proteomic tree based on the RNA polymerase amino acid sequence was produced showing vTRX32-1 and it’s relatives in the context of other phages representing major *Autographiviridae* clades. Both protein and nucleotide sequences were produced in MEGAX using MUSCLE (following Kumar et al., 2015) using a maximum likelihood method (Tamura-Nei model), with 1,000 bootstrap replications.

Phage mobilization within bacterial lineages was assessed using empress (Santichaivekin et al., 2020) maximum parsimony reconciliations. Reduced phylogenies were produced as above using the 9 prophages and corresponding lysogen strains. Cost weightings for events were set as follows: cospeciation = 0, transfers = 1, duplications = 2 and loss = 1–2. Analysis of the “cost space” (the impact of cost values on the predicted outcomes) showed that outcomes were robust to changes in the weighting of duplication events. A value of 2 was chosen as all prophages in this group show integration into one conserved site, making duplication unlikely. The relative weighting of transfer and loss is less clear and strongly affects the outcome therefore loss was considered at two probability levels, firstly with equal probability with transfer (loss = 1) and secondly, as less likely than transfer (loss = 2). Where loss = 1, 20 maximum parsimony reconciliations (MPRs) were estimated and where loss = 2, 38 MPRs were estimated. These were clustered into 2 and 4 groups of MPRs, respectively, and a representative scenario of each cluster produced following program guidance.

## Results and Discussion

### The vTRX32-1 Phage Family

**FIGURE 1:**
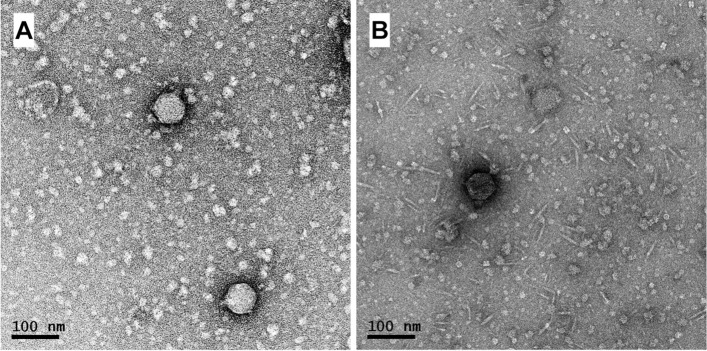
Morphology of phage vTRX32-1 and RHEph01 shown by transmission electron microscopy. vTRX32-1 **(A)** follows the morphology of previously described RHEph01 **(B)**.

The vTRX32-1 phage family represents a novel group of temperate phages isolated within *Rhizobium leguminosarum* bv. *trifolii* (*Rlt)* strains. Originally, vTRX32-1 (vB_RleA_TRX32-1) was isolated from strain TRX32 (Kumar et al., 2015) (from a clover nodule in York, United Kingdom), sequenced and visualized by transmission electron microscopy (Figure 1). Both sequence and morphology place this phage as a member of the *Autographiviridae*—related to *Podoviridae*. Subsequent screening of *Rlt* published sequences revealed four further lysogens containing closely related prophages; strains SM48 and SM135A (isolated from white clover nodules in Denmark) and SM95 and SM113 (isolated from white clover nodules in Rennes, France) (Cavassim et al., 2020; Supplementary Table 1). All five *Rlt* phages could be isolated by induction and formed plaques on isolating strain *Rlt* TRX19.

Blast searches revealed that these prophages were also related to the previously described lytic phage, RHEph01, isolated on *R. etli* in Mexico (Santamaría et al., 2014) as well as four further putative prophages in more distantly related *Rhizobium* strains; *R. anhuiense* CCBAU 23252, *R. loessense* CGMCC 1.3401, *Rhizobium* sp. NXC14 and *Rhizobiales* sp. AFS092257 (Supplementary Table 1).

Patterns of relatedness between phages show that 8 phages—five *Rlt* phages, the NXC14 and CCBAU 23252 putative prophages and the RHEph01 lytic phage—form a clade of > 70% average nucleotide identity (ANI) (Supplementary Figure 1). RHEph01 is currently classified by ICTV as the sole member of the genus *Paadamvirus*, in the family *Autographiviridae* (International Committee on Taxonomy of Viruses [ICTV], 2021) and under current guidance this places these 7 additional phages within this genus. Within this group, the *Rlt* phages form a distinct, more closely related cluster (ANI 87.3–93.9%; Supplementary Figure 1). Under classification guidelines, however, these phages would each correspond to a separate species (< 97% ANI) (International Committee on Taxonomy of Viruses [ICTV], 2021).

### Variable Life Histories Across the vTRX32-1 Like *Rlt* Phages

**FIGURE 2:**
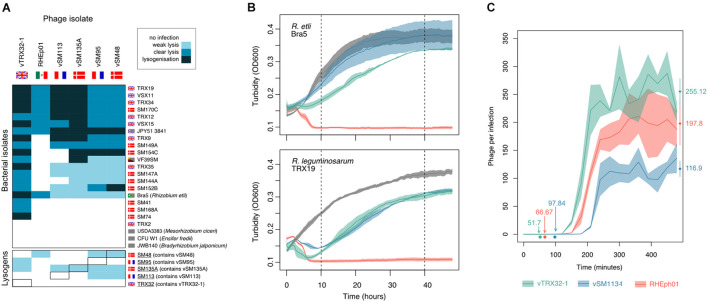
Phage growth characteristics. **(A)** Infection host range. Six phages were challenged against bacterial strains from the same isolation sites as well as type strains of more distantly related Rhizobia. Strain origin is denoted by flag with gray blocks indicating lab strains. Bacterial strains in the left-hand box are the original lysogenic hosts. Infectivity was graded as weak lysis (turbid clearing in high titer spots), clear lysis (clear high titer spots and individual plaques at low dilutions), and lysogenization (clear high titer spot at day 1, subsequently becoming turbid). **(B)** Phage virulence effects on growth of two *Rhizobium* hosts, *R. etli* Bra5 (upper panel) and *R. leguminosarum* TRX19 (lower panel). The turbidity of bacterial cultures containing phage at 10:1 bacterial:phage ratio was observed overtime. Virulence (lysis) was estimated from reduction in bacterial growth relative to the phage free control at 10 h post-infection and overall impact of phages (including lysis and recovery) measured at 40 h post-infection. Lines shown are averages across 3 replicates with area denoting standard error. **(C)** Lytic phage reproduction rate from bacterial infection. Phage replication was assayed on *R. leguminosarum* host TRX19 using the one-step growth curve method. For each phage latent time (i.e., incubation period) and burst size (number of phages produced per infection) were estimated from growth curve analysis and shown and round points and diamond points, respectively (*n* = 4).

Analysis of phage infection dynamics showed significant variation within this small group. The infectious host ranges of the five *Rlt* phages and the lytic phage RHEph01 were compared on a panel of natural isolates drawn from the same *Rlt* communities as vTRX32-1 and the vSM phages as well as several type strains from more distantly related species (Figure 2A). All 6 phages were able to infect a wide range of *R. leguminosarum* hosts as well as *R. etli* strain Bra5 indicating a relatively broad host range, i.e., spanning multiple *Rhizobium* species (Ross et al., 2016). No phages were able to infect strains from more distantly related genera. Within the host panel (primarily *Rlt* strains) phage RHEph01 had the most narrow host range—which very likely reflects local adaptation of the *Rlt* phages to European *R. leguminosarum* populations, compared with RHEph01 which was isolated in Mexico where *R. leguminosarum* is less dominant. RHEph01 showed no evidence of lysogeny in either species, confirming the findings of Santamaría et al. (2014) that this phage is purely lytic, at least under lab conditions. All five *Rlt* phages were able to form lysogens in new hosts, though lysogens were not detected for all hosts. Phage vTRX32-1 was by far the most infectious. Indeed, relatively few resistant *R. leguminosarum* isolates have been identified. The four vSM phages had a slightly reduced host range with many infections that produced zones of clearing but no visible individual plaques, designated as weak lysis. These phages divided into two patterns of host range—with vSM113 and vSM135A showing more evidence of lysogeny of strains compared to vSM95 and vSM48. These patterns do not follow either phage relatedness (Supplementary Figure 1) nor isolation environment, e.g., vSM95 and vSM113 were isolated from the same site in France yet show different patterns of infection. Multiple phages also showed weak but consistent lysis of lysogen strains containing either related phages or, in the case of vSM48 and vSM135A, their own lysogens. This may imply that superinfection immunity is impaired, or that the zone of growth inhibition could be associated with induction triggered by high rates of secondary phage adsorption (Abedon, 2019).

Phage virulence was assessed for 3 phages—vTRX32-1 (isolated as a prophage in United Kingdom), vSM113 (isolated as a prophage in France) and RHEph01 (isolated as a lytic phage in Mexico)—on growing cultures of mutually susceptible *R. leguminosarum*, TRX19, and *R. etli*, Bra5, hosts. At 10 h, where growth is most severely affected by phage killing (Figure 2B), virulence varied significantly between phages (*F*_2_, _12_ = 10.736, *p* = 0.00212) but not bacterial host (*F*_1_, _12_ = 4.005, *p* = 0.0685). All phages reduced host density at this time point but lytic phage RHEph01 was significantly more virulent than the two *Rlt* phages (contrasts_*vSM*__113__—__*RHEp*__01_: *t* = –4.352, *p* = 0.0025, contrasts_*vTRX*__3__2__–__1__—__*RHEp*__01_: *t* = –3.067, *p* = 0.0245). No significant difference was observed between these two *Rlt* phages (contrasts_*vSM*__113__—__*vTRX*__3__2__–__1_: *t* = 1.219, *p* = 0.465).

Following this initial drop in bacterial density in phage containing populations, bacteria infected with either of the *Rlt* phages recovered dramatically (Figure 2B). Populations infected with RHEph01, however, experienced no such rescue. Correspondingly, reduction in bacterial growth at 40 h differed significantly between phages (*F*_2_, _12_ = 84.791, *p* < 0.0001), which was again driven by difference between RHEph01 and the *Rlt* phages (contrasts_*vSM*__113__—__*RHEp*__01_: *t* = –11.683, *p* < 0.001; contrasts_*vTRX*__3__2–1__—__*RHEp*__01_: *t* = –10.303 *p* < 0.001) with no difference between the vSM113 and vTRX32-1 (contrasts_*vSM*__113__—__*vTRX*__3__2–1_: *t* = 1.309, *p* = 0.417). PCR of colonies isolated from a subset of strains confirmed that recovered bacteria infected with both *Rlt* phages had become lysogens. In contrast RHEph01 effectively cleared the population without any appearance of resistance emerging, again confirming that it is unable—or unlikely—to form lysogens.

The dynamics of lytic phage replication were further analyzed using a one step growth curve of these three strains on the mutually susceptible host TRX19. In comparison with other phages, the *Rlt* phages have relatively long latent periods and large burst sizes. The latent period—the time between adsorption and lysis when lytic phage particles are assembled—was relatively long; from 50 min in vTRX32-1 to over 90 min in the case of vSM113. In comparison, in *E. coli* phages latent periods can be as short as 20 min (Topka et al., 2018). All 3 phages were able to replicate as lytic phages on the host and produced large burst sizes of up to 255 ± PFUs per cell in the case of vTRX32-1 (Figure 2C). Phages differed significantly in both growth characteristics (burst size: *F*_2_, _9_ = 6.522, *p* = 0.018, latent period: *F*_2_, _9_ = 38.21, *p* < 0.0001). vSM113 exhibited the least efficient lysis—both in terms of longest latent period and smallest burst size—while vTRX32-1 exhibited the most productive infections with burst size being significantly larger compared with vSM113-1 (contrasts_*vTRX*__3__2–1__—__*vSM*__11__3–1_; *t* = 3.594, *p* = 0.014) and latent period shorter than both phages (contrasts_*vTRX*__3__2–1__—__*vSM*__11__3–1_; *t* = –8.567, *p* < 0.001, contrasts_*vTRX*__3__2–1__—__*RHEph*__01_: *t* = –2.781, *p* = 0.051).

### Comparative Genomics of the vTRX32-1 Like Phages and Wider Relatives

In addition to the five vTRX32-1-like *Rlt* phages and RHEph01, we identified four related prophages within published sequences of different *Rhizobium* species and included these putative phages for a comparison of the wider clade. Initial comparison between phage genomes shows that genome architecture across this group of 10 phages is well conserved; they are between 42 and 45 kb long, have an average GC content of 58.2–59.5% and contain between 52 and 55 predicted open reading frames each. In the vTRX32-1 genome 25 genes were annotated, 23 of which were present in all close relatives. tRNA-SCAN-SE identified a single tRNA-Leu in all pro/phage genomes, and additional tRNAs in 4 of the 10 genomes.

All prophages share the same attachment site. A conserved terminally repeated 12 bp motif (“CAGATTTAGGTT”) was identified at the terminal ends of all prophages—annotated by PHASTER as the attL/attR sites. Phages sequenced in their circular form, vTRX32-1 and RHEph01, both carry this repeated motif in the same position. In *R. leguminosarum* strains which lack the prophage this 12 bp motif is present within a tRNA-Leu indicating that this is the site of prophage integration (attB; see Supplementary Figure 2). tRNA sequences are used by a range of other mobile genetic elements for site-specific integration (Reiter et al., 1989), including other rhizophages (Semsey et al., 2002; Halmillawewa et al., 2016), retrotransposons (Mularoni et al., 2012), and cyanopodoviruses (Sullivan et al., 2005). This includes closely related pelagibacter viruses in the *HTVC019Pvirus* genus (Zhao et al., 2019). The tRNA-Leu carried within the phage genome lacks the 12 bp motif, presumably replacing function of the chromosomal tRNA-Leu interrupted through integration but preventing “super-integration”—integration of additional phages into the phage itself. It is common for tRNA integrating phages to encode a replacement tRNA, although this is more often in the form of a partial tRNA sequence which completes one half of the disrupted gene (Halmillawewa et al., 2016).

**FIGURE 3:**
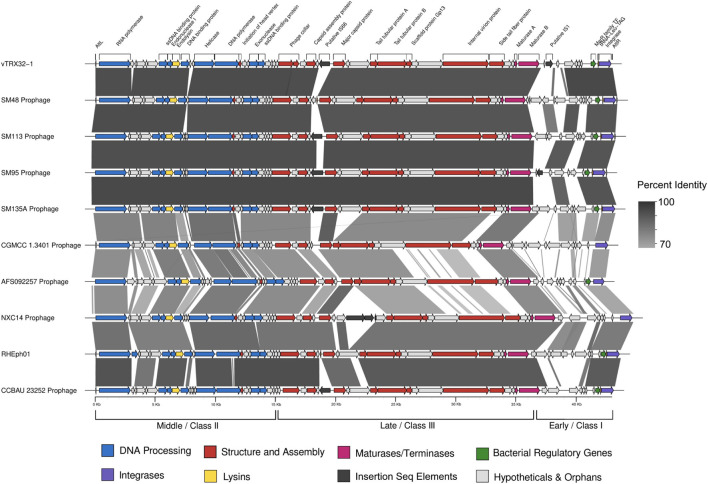
Genome comparison of vTRX32-1 and related phage/prophage. Blastn hits are represented by gray bars, with darker shades of gray indicating higher percent identity. Genomes are organized from top to bottom based on the phage phylogeny. Genes are colored according to their functions.

Genome analysis revealed that this group of *Rhizobium* phages have compact genomes typical of the *Autographiviridae* and related (and better described) *Podoviridae*, with similar genome size, synteny and core gene content (Figure 3). As is typical for mobile genetic elements such as prophages, roughly half of genes are of unknown function. Distribution of hypothetical genes as well as genome conservation was uneven across the genome. Based on homology to type phage, *E. coli* bacteriophage T7, genes can be grouped into early, middle and late clusters—named on the basis of their transcription order (also referred to as the class I, II and III genes) (Dunn and Studier, 1983; Lindell et al., 2007). Regions of the genome showing homology to genes from middle and late regions, involved in DNA processing and phage structure, respectively, are characterized by high levels of synteny and nucleotide homology. Genes in these regions contain predominantly phage-like predicted promoters and can be well annotated by homology with other known core phage genes.

**FIGURE 4:**
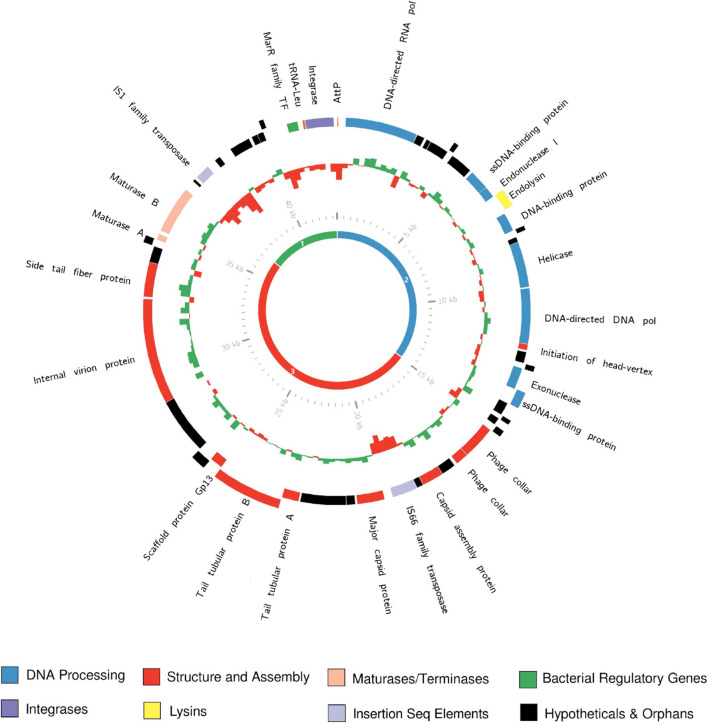
The annotated genome of vTRX32-1. The outer ring shows ORFs predicted by RAST, with colors corresponding to their predicted functions. The inner histogram represents GC%, GC% above the genome average is green and GC% below the average is red. Histogram size indicates the disparity between the GC% at that locus (250 bp bins) and the genome average GC%. The central ring shows the location of the 3 gene classes in the vTRX32-1 genome (green, blue, and red corresponding to early, middle, and late, respectively).

In contrast, the region downstream of the maturase B gene, which contains genes with homology to early region genes, is highly variable in both nucleotide identity (Figure 3) and ORF presence/absence (Supplementary Figure 3). The majority of ORFs called within this region are of unknown function, based on both homology to published sequences or identifiable domains. In contrast to middle and late genes, predicted promoters for this region were typical of bacteria, rather than phage genes. Finally, the early region also has significantly lower GC% (54.32 ± 0.65%) than the rest of the genome (59.95 ± 0.24%), (*t*_9_ = 29.67, *p* < 0.00001; Figure 4), characteristic of genes recently acquired by horizontal gene transfer. Early region genes, as the name implies, are transcribed first upon entry to the cell. In closely related phages, such as T7, these genes encode proteins that must act fast to overcome host defenses and takeover host replicative machinery (Studier, 1972; Dunn and Studier, 1983). Thus, variation in this region could reflect selection for host specificity and/or infectivity driving divergence in this region, which has been shown to drive recombination between phages (De Paepe et al., 2014). This pattern of conservation and variability is common amongst close relatives within the *Autographiviridae* (Zhu et al., 2010; Labrie et al., 2013; Zhao et al., 2013; Hamdi et al., 2016; Kawasaki et al., 2016).

### Evidence of Horizontal Gene Transfer Within Phage Genomes

Phage genomes often feature significant mosaicism (Juhala et al., 2000) and are themselves subject to integration of other mobile elements. Three insertion sequences, in the form of transposases, were identified across the 10 phages. Firstly, a putative IS66 element (Machida et al., 1984) was found in 4/5 of the *Rlt* phages, as well as a putative prophage detected in *R. anhuiense* CCBAU23252. IS66 elements were originally isolated in the *Agrobacterium tumefaciens* [now reclassified as *Rhizobium radiobacter* (Young et al., 2001)] Ti plasmid (Machida et al., 1984), and are common throughout *Rhizobium* species (Rochepeau et al., 1997; González et al., 2006). In addition to IS66 elements both the vTRX32-1 and vSM95-2 genomes contain an additional gene annotated as an IS1 transposase within the variable early region. Finally, an IS21 transposase is present in the genome of a putative prophage identified in *Rhizobium* spp. NXC14. This IS element is identical to IS21 transposase domains found on NXC14 plasmids, pRspNXC14a and pRspNXC14c, as well as being closely related to other insertion sequences on a variety of *Rhizobium* replicons, making it likely that it was acquired from co-habiting plasmids. Like the early region genes, these regions are notable for their low GC% content (Figure 4). IS elements are known to be highly active within *Rhizobium* (Hernandez-Lucas et al., 2006), and are thought to contribute to *Rhizobium* genome evolution, creating their characteristic mosaic structure (González et al., 2003; Hernandez-Lucas et al., 2006). The presence of IS elements within phage genomes suggests that prophages can act as a conduit for dissemination of IS elements throughout *Rhizobium.*

In contrast to plasmids where they are common, the presence of insertion sequences is unusual in phages, and in temperate phages specifically (Leclercq and Cordaux, 2011). One survey of phage genomes found 92% of active temperate phages (i.e., excluding immobile/partial prophages) had no detectable insertion elements (Leclercq and Cordaux, 2011). This is likely due to the strict genome size constraints imposed by phage capsid size, the density of essential genes in phage genomes, and the huge population sizes of active phages resulting in a strong selection pressure against insertion element retention. In the *R. leguminosarum* phages, for which lysogen strains were available, the presence of insertion elements does not impair the ability of the phages to produce functional infective phage particles as all prophages tested could be induced. In addition to the IS66 element phages vTRX32-1 and vSM95-1 also carry an additional transposase, yet both have been shown to form efficient lytic infections, suggesting the phages are capable of accommodating additional novel DNA into their genome without impairing the packaging of phage DNA.

In addition to these insertion elements, four of the 10 phages carry unique genes that do not appear on other phages in the clade suggesting they have been acquired by horizontal gene transfer. Interestingly these genes are found in the same location—directly downstream of the RNA polymerase gene. In two cases functional information indicates that these additional genes may be adaptive. Firstly, the lytic phage RHEph01 contains a Bro-domain putative antirepressor. Antirepressors are involved in prophage lysis-lysogeny decisions, which may provide an explanation for the lytic nature of this phage. When integrated into the bacterial genome, most prophages express a repressor protein which silences phage gene transcription and prevents the phage excising, producing infectious virus particles and lysing the host (Bednarz et al., 2014). Antirepressors, such as Bro domain proteins represent one mechanism for relieving repression of phage genes when entering lysis (Lemire et al., 2011) and have been identified in numerous prophages (Botstein et al., 1975; Farlow et al., 2018). Interestingly, however, the absence of this gene on the 9 other phage genomes suggests that it has been acquired independently and is not required to moderate the lysogeny-lysis switch. While antirepressors are typically associated with integrated prophages exiting lysogeny, it is possible that the presence of this gene could explain the inability of RHEph01 to form stable lysogenic infections. For example, overactive expression of the antirepressor gene may inhibit repressor activity entirely, preventing stable lysogeny from establishing. Further work will be needed to confirm the role of this gene in the conversion of phage RHEph01 to a purely lytic lifestyle.

Secondly *Rhizobiales* sp. AFS092257 prophage contains a putative cytosine-methyltransferase. Orphan DNA-methyltransferases have been identified on numerous phage genomes and have been linked to protection against host restriction enzymes used to defend hosts from phage infection (Murphy et al., 2013, 2014). DNA-methyltransferases methylate restriction sites within the phage genome, preventing host restriction enzymes from cleaving phage DNA (Murphy et al., 2013, 2014). For example a methyltransferase encoded on the genome of the *Bacillus subtillis* prophage SPβ becomes active after the prophage is induced, methylating newly replicated phage DNA at BsuR restriction sites and as a result protecting them from the BsuR endonuclease (Trautner et al., 1980). It may be that the AFS092257 prophage cytosine-methyltransferase performs a similar protective function.

Finally vTRX32-1, and the prophage identified in *R. anhuiense* CCBAU23252 each carry a unique ORF at the same site as these two genes, with no homology to each other and no functional domains which could be identified. The complete lack of homology between genes at this site suggest this location is a hotspot for gene gain and loss.

### Relatedness and Evidence for Transfer of *Rhizobium* Phages

**FIGURE 5:**
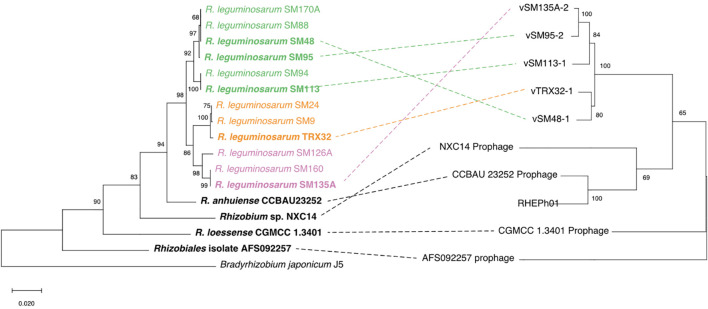
The phylogenies vTRX32-1-like phages and their bacterial hosts. The phage phylogeny (right) was produced using the concatenated nucleotide sequence of 5 conserved genes (see section “Materials and Methods”). The bacterial phylogeny (left) is based on the nucleotide sequence of the housekeeping gene *recA*. The colored highlights group *Rhizobium leguminosarum* strains into their respective genospecies. Lysogenic strains are shown in bold and dotted lines link prophage to their bacterial hosts. Branch lengths indicate substitutions per site for both phylogenies (scale shown in bottom left). Numbers by each clade indicate the percent of bootstrap replicates that support each clade.

Patterns of relatedness between phages were estimated using a 5-concatenated-gene phylogeny and compared to their host strain phylogeny (Figure 5). The phage phylogeny based on core genes follows the general pattern observed from ANI calculated across the whole phage genomes (Supplementary Figure 1), with prophages isolated from *Rlt* strains (vTRX32-1, vSM48, vSM95, vSM113, and vSM135A) being most closely related to each other and the five additional phages more distantly related. Within the host tree 7 additional *Rlt* strains were included which lack any prophages at the attB site demonstrating that lysogenic strains are nested within closely related but non-lysogenic strains. Indeed, the four SM-strain lysogens (Cavassim et al., 2020) were identified within a wider strain collection of 196 fully *Rlt* sequenced strains. Detailed assessment of these genomes (through both blastn and inspection of the highly conserved insertion site) found no intact or degraded vTRX32-1-like phages and attB insertion sites were intact.

**FIGURE 6:**
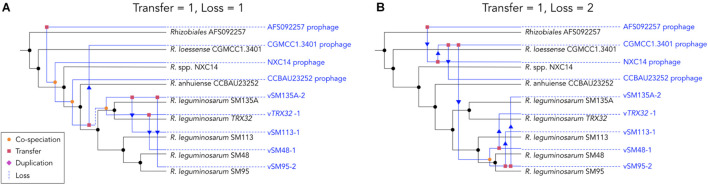
Maximum parsimony reconciliations for prophage and lysogen phylogenies. Trees show a randomly selected example of possible evolutionary events explaining the discordance between phylogenies under two sets of cost parameters. **(A)** Shows one of 20 possible reconciliations where loss is equal to transfer (loss = 1, transfer = 1). **(B)** Shows one of 38 possible reconciliations where loss is less likely than transfer (loss = 2, transfer = 1). Host trees are shown in black while estimated phage trees are shown in blue.

Analysis of co-phylogeny was performed for the 9 prophage and lysogen matched pairs using empress (Santichaivekin et al., 2020), which calculates maximum parsimony reconciliations (MPRs) for patterns of relatedness between 2 groups of species (e.g., hosts and symbionts). Relative costs of four possible evolutionary events were set as: cospeciation = 0, transfer = 1, duplication = 2 and loss was modeled at two levels, 1 and 2. Where the likelihood of loss events was equal to transfer events (loss = 1) reconstruction estimated that the pattern of phage distribution within the host phylogeny could be explained by 4 cospeciation events, 2 losses and 4 transfer events (Figure 6A). Where loss events are less likely than transfer events (loss = 2) reconstruction estimates that the pattern of distribution is predominantly explained by transfers events (7) rather than cospeciation events (1) (Figure 6B). Under both scenarios the distribution of phages and hosts is not significantly different from that of random trees (loss = 1, *P* < 0.168, loss = 2, *P* = 0.693), which indicates that processes such as transfer rather than simple cospeciation is the dominant process explaining phage distribution. This is supported by the relative rarity of phages in closely related populations and lack of evidence for degraded phage genomes within the insertion site.

**FIGURE 7:**
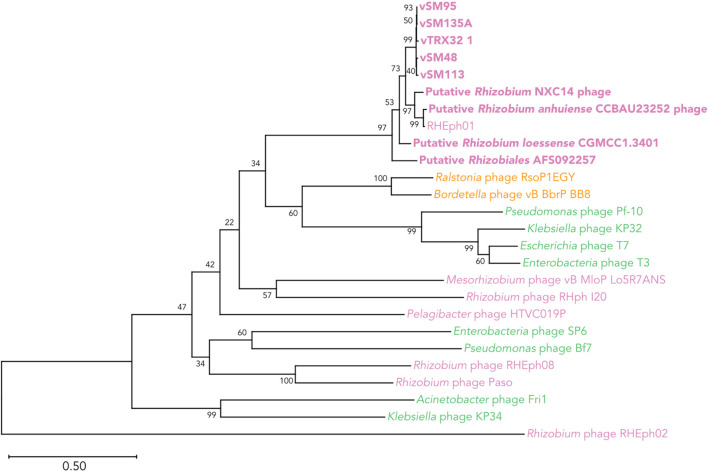
Phylogenetic relationship of the TRX32-1 like phages in and the wider *Autographiviridae* clade based on DNA-dependent RNA Polymerase protein sequences. The vTRX32-1-like phages are shown in bold along with representative examples of the larger *Autographiviridae* clade. Label color indicates the class of host bacteria for each phage; pink = Alphaproteobacteria, yellow = Betaproteobacteria, green = Gammaproteobacteria. Numbers by each clade indicate the percent of bootstrap replicates that support each clade.

Comparison of phage RNA polymerase sequences places the vTRX32-1-like group within the newly demarcated family *Autographiviridae*, previously a subfamily of the *Podoviridae* (Walker et al., 2019). Phages from this clade infect a wide diversity of bacteria spanning Alphaproteobacteria (including *Rhizobium*), Betaproteobacteria, and Gammaproteobacteria (Figure 7).

## Conclusion

The vTRX32-1 like phages are a novel group of temperate phages infecting *Rhizobium leguminosarum*, closely related to the previously described lytic phage RHEph01—suggesting this phage is ancestrally temperate. In addition we identified a wider group of phage genomes within other Rhizobium species indicating that these phages form part of a group of temperate phages which have a long evolutionary history within *Rhizobium* spp.

Phenotypic demonstrate that the vTRX-1-like phages are inducible, widely infectious, able to form new lysogenic partnerships. Intriguingly, no degraded versions of these phages were identified within 192 other *Rlt* strains suggesting both that they are being maintained by active mobilization and are not under selection for degradation and loss of function at least in these populations. This supports the findings from the phylogenetic distribution of the wider phage group, which implies a significant contribution of horizontal transmission to phage distribution over longer evolutionary timescales. We also identified instances of horizontal gene transfer within the phage genomes including insertion sequences—which are known to play a role in *Rhizobium* evolution—and several unique genes which are mostly likely to have been acquired following divergence between phages.

Temperate phages are relatively unstudied in terrestrial species such as *Rhizobium*, with very few published examples (Halmillawewa et al., 2016). In both pathogenic and marine ecosystems temperate phages play a significant role in bacterial ecology and evolution. The presence of this active group of temperate phages suggests they may have a role to play in Rhizobia communities.

## Data Availability Statement

The datasets presented in this study can be found in online repositories. The names of the repository/repositories and accession number(s) can be found below: https://www.ncbi.nlm.nih.gov/genbank/, MW023914.1.

## Author Contributions

SM and EH contrived and conducted experiments and analysis for the study. PY, SM, and RS provided strains and advised on analysis. EH awarded the funding. All authors contributed to the preparation of the manuscript.

## Conflict of Interest

The authors declare that the research was conducted in the absence of any commercial or financial relationships that could be construed as a potential conflict of interest.

## Publisher’s Note

All claims expressed in this article are solely those of the authors and do not necessarily represent those of their affiliated organizations, or those of the publisher, the editors and the reviewers. Any product that may be evaluated in this article, or claim that may be made by its manufacturer, is not guaranteed or endorsed by the publisher.

## Footnotes

1
https://phaster.ca

2
http://rhea.icbm.uni-oldenburg.de/VIRIDIC

## Supplementary Material

Supplementary Figure 1 VIRIDIC genome comparisons for *Rhizobium* phages. Grid shows pairwise comparisons for each of the 10 phage genomes. Squares in the top right show ANI values for whole phage genomes with intensity of color denoting intergenomic similarity. Squares in the bottom left half show the genome length ratio (numbered) and the proportion of sequence aligned for each comparison (shading). Highlighted squares represent the 5 phages isolated from *Rhizobium leguminosarum* (green box) and the phages which share > 70% ANI, and are therefore likely members of the *Paadmavirus* genus (pink).

Supplementary Figure 2 Illustrating the integration of the vTRX32-1 like phages into the *Rhizobium* genome (based on genome comparisons of vTRX32-1 and vRHEph01 with closely related prophage). The tRNA-Leu in the *Rhizobium* genome contains a 12 bp motif also carried by the phage (red), this acts as the region of homology (attB/attP site) for phage integration. In the process of integration the bacterial tRNA-Leu (yellow) is interrupted and replaced by a phage encoded tRNA-Leu (yellow, hashed) in the phage genome.

Supplementary Figure 3 Percent identity between amino acid sequences predicted by RAST in the vTRX32-1 genome and those of related phage/prophage. Genes (*X*-axis) are ordered from the highest percent identity (left) to the lowest (right), phage/prophage genomes (*Y*-axis) are ordered similarly from top to bottom. Colored highlights adjacent to gene names indicate predicted class membership (yellow = early/class I, blue = middle/class II, red = late/class III). Pairwise blastP was used to generate percent identity values and only hits with an *E*-value less than 1E-4 were included.

Supplementary Table 1 Details of phages and putative phages identified as related to vTRX32-1.

Supplementary Table 2 Annotated data sets from the 3 phenotypic assays; phage virulence, phage replication dynamics (one step growth curve) and host range. Details are included in each dataframe.

Supplementary Data Sheet 1 Bioinformatics pipeline for phage genome assembly and provisional annotation.
