# Supplementary figures and images for: Introducing a Novel, Broad Host Range Temperate Phage Family Infecting Rhizobium leguminosarum and Beyond
Source: Front Microbiol. 2021 Nov 9;12:765271. doi: 10.3389/fmicb.2021.765271 (PMC8631192; doi:10.3389/fmicb.2021.765271)

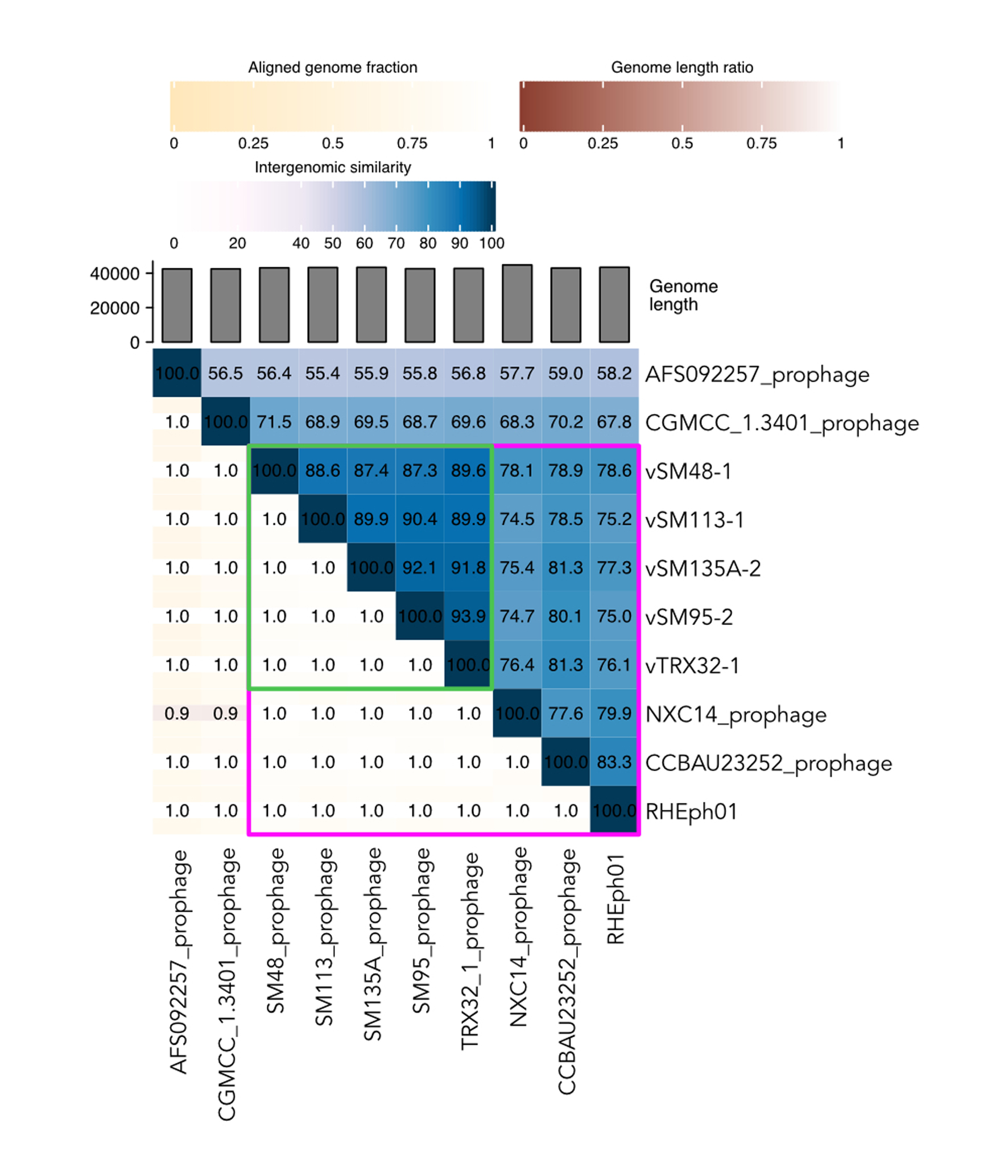

Supplement: Supplementary Figure 1 — VIRIDIC genome comparisons for Rhizobium phages. Grid shows pairwise comparisons for each of the 10 phage genomes. Squares in the top right show ANI values for whole phage genomes with intensity of color denoting intergenomic similarity. Squares in the bottom left half show the genome length ratio (numbered) and the proportion of sequence aligned for each comparison (shading). Highlighted squares represent the 5 phages isolated from Rhizobium leguminosarum (green box) and the phages which share > 70% ANI, and are therefore likely members of the Paadmavirus genus (pink). [file Image_1.JPEG]

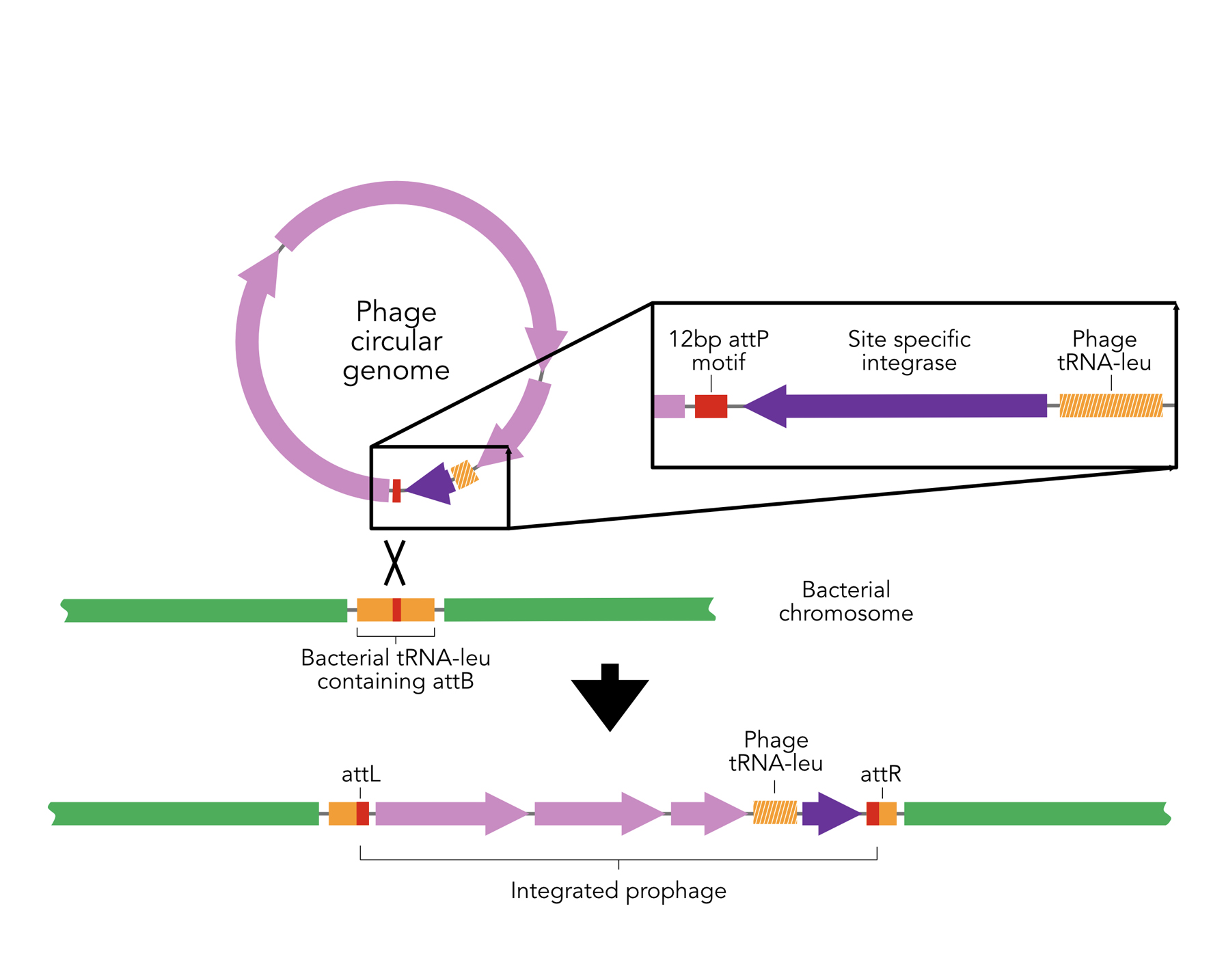

Supplement: Supplementary Figure 2 — Illustrating the integration of the vTRX32-1 like phages into the Rhizobium genome (based on genome comparisons of vTRX32-1 and vRHEph01 with closely related prophage). The tRNA-Leu in the Rhizobium genome contains a 12 bp motif also carried by the phage (red), this acts as the region of homology (attB/attP site) for phage integration. In the process of integration the bacterial tRNA-Leu (yellow) is interrupted and replaced by a phage encoded tRNA-Leu (yellow, hashed) in the phage genome. [file Image_2.JPEG]

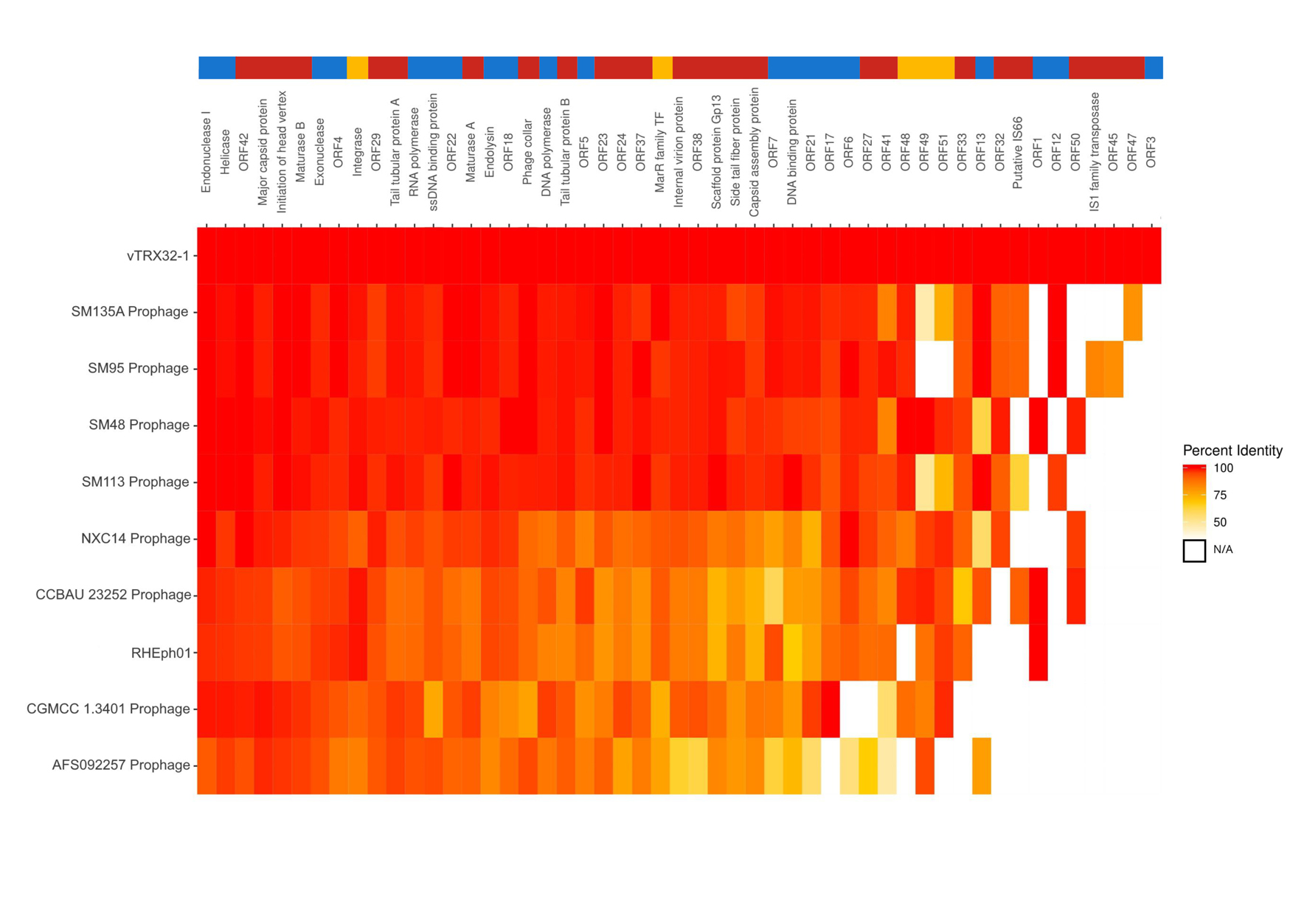

Supplement: Supplementary Figure 3 — Percent identity between amino acid sequences predicted by RAST in the vTRX32-1 genome and those of related phage/prophage. Genes (X-axis) are ordered from the highest percent identity (left) to the lowest (right), phage/prophage genomes (Y-axis) are ordered similarly from top to bottom. Colored highlights adjacent to gene names indicate predicted class membership (yellow = early/class I, blue = middle/class II, red = late/class III). Pairwise blastP was used to generate percent identity values and only hits with an E-value less than 1E-4 were included. [file Image_3.JPEG]
