# Supplementary material for: Introducing a Novel, Broad Host Range Temperate Phage Family Infecting Rhizobium leguminosarum and Beyond
Source: Front Microbiol. 2021 Nov 9;12:765271. doi: 10.3389/fmicb.2021.765271 (PMC8631192; doi:10.3389/fmicb.2021.765271)
Supplement: Supplementary Data Sheet 1 — Bioinformatics pipeline for phage genome assembly and provisional annotation. [file Data_Sheet_1.docx]

Pipeline for assembly of Phage genome from illumina reads

# -------------------- PREP - getting the right programmes --------------------

# Make sure homebrew is installed and up to date

brew update

brew tap brewsci/bio # point it towards a directoy of useful stuff

#check what you have in your secret bin folder

echo $PATH # reveals all the PATH folder on the computer then you can....

ls /usr/local/bin # (this is my file path - if it doesn't work check output above) should list all the things in the bin folder where programs are stored.

# You will need:

sickle, fastqc, spades, bwa, samtools, bcftools

# install what youre missing

brew install sickle # read trimming

brew install fastqc # read quality checking (NB. I needed to update Xcode to allow dependancies to install first - xcode-select --install)

brew install spades # assembly

brew install bwa # aligns reads

# brew install bowtie2 # another aligner

brew install samtools # does lots of things! important for indexing and picking varients

brew install bcftools # makes files for varient calls

brew install blast

#QUAL=TOOLKIT/qualimap_v2.2.1/qualimap.jar

# -------------------- 1. Set up file paths --------------------

# specify some file names to make the code shorter. Comes into its own when doing multiple samples etc as you can just change ID here OR make a loop so it cycles trhough multiple files!

cd Google\ Drive/Sheffield/HarriLab/Phage_genome # the google drive is a bit annoying as the space in the file name causes too many issues. so just move into the phage genome file to work in using the cd command

SAMPLE_ID = S10

# set up output folders for the analysis. Useful to use the date as a unique identifier...

mkdir "$SAMPLE_ID"_alignment_*DDMMYY*

ROOT="$SAMPLE_ID"_alignment_*DDMMYY*

mkdir $ROOT/Fastqc_output

mkdir $ROOT/Trmd_reads

# -------------------- 2. Provisonal alignment --------------------

# QC reads

fastqc Reads/"$SAMPLE_ID"_1.fq.gz Reads/"$SAMPLE_ID"_2.fq.gz --outdir=$ROOT/Fastqc_output

# have a look at the html files for a representation of the quality. you can see that read qual really tails off at the end of the longer reads - as we would expect.

# trim reads using sickle

# sickle trims a read at the point needed to maintain an average quality specified by -q (default is 20) and removes reads shorter than the length specified by -l (default is 20bp)

sickle pe -f Reads/"$SAMPLE_ID"_1.fq.gz -r Reads/"$SAMPLE_ID"_2.fq.gz -o $ROOT/Trmd_reads/"$SAMPLE_ID"_1_trim.fq -p $ROOT/Trmd_reads/"$SAMPLE_ID"_2_trim.fq -s $ROOT/Trmd_reads/"$SAMPLE_ID"_singles.fq -t sanger -q 30 -l 100

# QC trimmed reads

fastqc $ROOT/Trmd_reads/"$SAMPLE_ID"_1_trim.fq $ROOT/Trmd_reads/"$SAMPLE_ID"_2_trim.fq $ROOT/Trmd_reads/"$SAMPLE_ID"_singles.fq --outdir=$ROOT/Fastqc_output

# Check that the quality has improved

# initial assembly using SPAdes - makes a rough de novo sequence

spades.py --only-assembler -1 $ROOT/Trmd_reads/"$SAMPLE_ID"_1_trim.fq -2 $ROOT/Trmd_reads/"$SAMPLE_ID"_2_trim.fq -s $ROOT/Trmd_reads/"$SAMPLE_ID"_singles.fq -o $ROOT/"$SAMPLE_ID"_assembly &

# -------------------- 3. Realignment --------------------

# move into the assembly folder

cd $ROOT/"$SAMPLE_ID"_assembly

# the contigs.fasta file contains the sequences assembled by SPAdes. you can see how many contigs there are with

grep -c \> contigs.fasta

# and list them with

grep \> contigs.fasta

# make a second copy (backup)

cp contigs.fasta "$SAMPLE_ID".fasta

# align! Align reads back to the rough assembly

# First make an index file **

bwa index -a is "$SAMPLE_ID".fasta

# then make the alignment, piping through SAM to BAM to sorted BAM

bwa mem -t 4 "$SAMPLE_ID".fasta ../Trmd_reads/"$SAMPLE_ID"_1_trim.fq ../Trmd_reads/"$SAMPLE_ID"_2_trim.fq > "$SAMPLE_ID".sam

samtools view -S -b "$SAMPLE_ID".sam > "$SAMPLE_ID".bam

samtools sort "$SAMPLE_ID".bam -o "$SAMPLE_ID".sorted.bam

#qualimap bamqc -bam "$SAMPLE_ID".sorted.bam #!!!! this is where I cant get qualimap to install. Will update when I figure it out.

done

# -------------------- 4. Contig selection --------------------

cd $ROOT/"$SAMPLE_ID"_assembly

SAMPLE_ID=S10

# Now we do another alignment - this time we select a contig (assuming here that large, well covered contigs are phages or parts of phages). We algin all the reads to just that contig so we have the best coverage (reads could have been algined to other random bits of sequence)

# if you had the qualimap results you would find a doc with lists of the contigs in the alignment and...

#

less "$SAMPLE_ID".sorted_stats/genome_results.txt

# would show you a bunch of stats and a list of the contigs. you would find that that the first (best coverage) is for a contic called.....

# NODE_1_length_42787_cov_29.276840

# pick out the largest contig (using 40,000 as the second contig is 37593)

samtools faidx "$SAMPLE_ID".fasta NODE_1_length_42787_cov_29.276840 > "$SAMPLE_ID"_C1.fasta

# align reads back to the big contig

bwa index -a is "$SAMPLE_ID"_C1.fasta "$SAMPLE_ID"_C1

bwa mem -t 4 "$SAMPLE_ID"_C1.fasta ../Trmd_reads/"$SAMPLE_ID"_1_trim.fq ../Trmd_reads/"$SAMPLE_ID"_2_trim.fq > S10_C1.sam

samtools view -S -b "$SAMPLE_ID"_C1.sam > "$SAMPLE_ID"_C1.bam

samtools view -S -b "$SAMPLE_ID"_C1.sam > "$SAMPLE_ID"_C1.bam

samtools sort "$SAMPLE_ID"_C1.bam -o "$SAMPLE_ID"_C1.sorted.bam

#qualimap bamqc -bam S10_C1.sorted.bam
